# Two functionally distinct kinetochore pools of BubR1 ensure accurate chromosome segregation

**DOI:** 10.1038/ncomms12256

**Published:** 2016-07-26

**Authors:** Gang Zhang, Blanca Lopez Mendez, Garry G. Sedgwick, Jakob Nilsson

**Affiliations:** 1 The Novo Nordisk Foundation Center for Protein Research, University of Copenhagen, Faculty of Health and Medical Sciences, Blegdamsvej 3B, 2200 Copenhagen, Denmark

## Abstract

The BubR1/Bub3 complex is an important regulator of chromosome segregation as it facilitates proper kinetochore–microtubule interactions and is also an essential component of the spindle assembly checkpoint (SAC). Whether BubR1/Bub3 localization to kinetochores in human cells stimulates SAC signalling or only contributes to kinetochore–microtubule interactions is debated. Here we show that two distinct pools of BubR1/Bub3 exist at kinetochores and we uncouple these with defined BubR1/Bub3 mutants to address their function. The major kinetochore pool of BubR1/Bub3 is dependent on direct Bub1/Bub3 binding and is required for chromosome alignment but not for the SAC. A distinct pool of BubR1/Bub3 localizes by directly binding to phosphorylated MELT repeats on the outer kinetochore protein KNL1. When we prevent the direct binding of BubR1/Bub3 to KNL1 the checkpoint is weakened because BubR1/Bub3 is not incorporated into checkpoint complexes efficiently. In conclusion, kinetochore localization supports both known functions of BubR1/Bub3.

## Abstract (web summary)

The BubR1/Bub3 complex regulates chromosome segregation to enable proper kinetochore-microtubule interactions and is also required for the spindle assembly checkpoint. Here the authors show that two distinct pools of BubR1/Bub3 exist at kinetochores to support both known functions of BubR1/Bub3.

## Graphical abstract

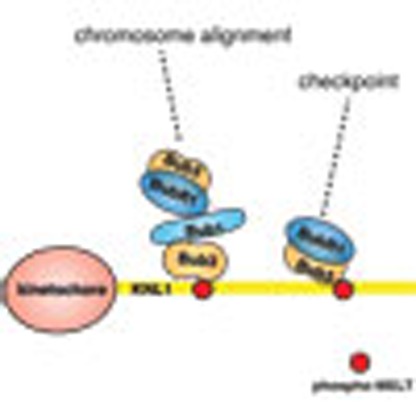

The spindle assembly checkpoint (SAC) ensures accurate chromosome segregation by delaying anaphase entry until all chromosomes align at the metaphase plate^1^,^2^. Anaphase entry depends on the activity of the anaphase-promoting complex/cyclosome (APC/C) bound to its co-activator Cdc20 and the SAC inhibits this complex in response to unattached kinetochores by generating a diffusible inhibitor referred to as the mitotic checkpoint complex (MCC)^3^. The MCC is composed of three checkpoint proteins, Mad2 and BubR1/Bub3 (referring to the complex of BubR1 and Bub3), bound stably to Cdc20 and this complex has the ability to bind and inhibit additional APC/C–Cdc20 complexes^4^,^5^,^6^,^7^. All checkpoint proteins accumulate and display rapid turnover at unattached kinetochores, suggesting that the MCC is generated locally there^8^,^9^,^10^. However, in addition to being an integral component of the MCC, BubR1/Bub3 also facilitates proper kinetochore–microtubule interactions by recruiting the PP2A–B56 protein phosphatase to kinetochores^11^,^12^,^13^,^14^,^15^. Whether BubR1/Bub3 only localizes to kinetochores to ensure kinetochore–microtubule interactions is debated due to conflicting results in the literature.

The checkpoint proteins Bub1 and BubR1 both bind stably to the Bub3 protein through a so-called GLEBS motif and this interaction is required for their kinetochore localization^16^,^17^,^18^,^19^. Bub1/Bub3 localizes by binding to one of multiple Met–Glu–Leu–Thr (MELT) repeats in the outer kinetochore protein KNL1 when the threonine residue is phosphorylated (MELTp) by the checkpoint kinase Mps1 (refs 20, 21, 22, 23, 24, 25). A conserved binding pocket on Bub3 provides the main interaction with MELTp and phosphorylation of an adjacent SHT motif can further strengthen the interaction with Bub3 in human cells^26^,^27^. In addition, BubR1/Bub3 kinetochore localization depends on a direct interaction between BubR1 and Bub1, which might be because the affinity of BubR1/Bub3 for MELTp is not enough to facilitate localization^28^,^29^. Surprisingly, preventing BubR1/Bub3 kinetochore localization by blocking Bub1 interaction increases checkpoint strength, but prevents stable kinetochore–microtubule attachment^28^,^29^. This and the fact that overexpressed cytoplasmic fragments of BubR1 can partially support the SAC have led to suggestions that BubR1/Bub3 kinetochore localization is not important for the checkpoint but instead contributes to checkpoint silencing through PP2A–B56 (refs 29, 30, 31, 32, 33). However, mutation of the GLEBS motif in BubR1 that abolish Bub3 binding prevents BubR1 kinetochore localization and strongly impairs checkpoint signalling. This could reflect a role of Bub3 bound to BubR1 in the checkpoint independently of localizing BubR1 to kinetochores^34^ or that the interaction of BubR1/Bub3 with kinetochores is not fully understood. Indeed, two kinetic distinct pools of BubR1 have been observed in fluorescent recovery after photobleaching experiments that are not readily explained by current models^9^.

Here we set out to further investigate the function of BubR1/Bub3 at kinetochores. We find that Bub3 localizes a pool of BubR1 onto kinetochores by directly binding MELTp repeats on KNL1, and that this pool of BubR1/Bub3 stimulates SAC signalling particularly when few unattached kinetochores are present. Instead, the major Bub1-dependent pool of BubR1/Bub3 supports chromosome alignment but not checkpoint signalling. We have thus uncovered two functionally distinct pools of BubR1 bound to KNL1 at kinetochores that combined ensures accurate chromosome segregation.

## Results

### A Bub1-independent pool of BubR1/Bub3 exists at kinetochores

**Figure 1:**
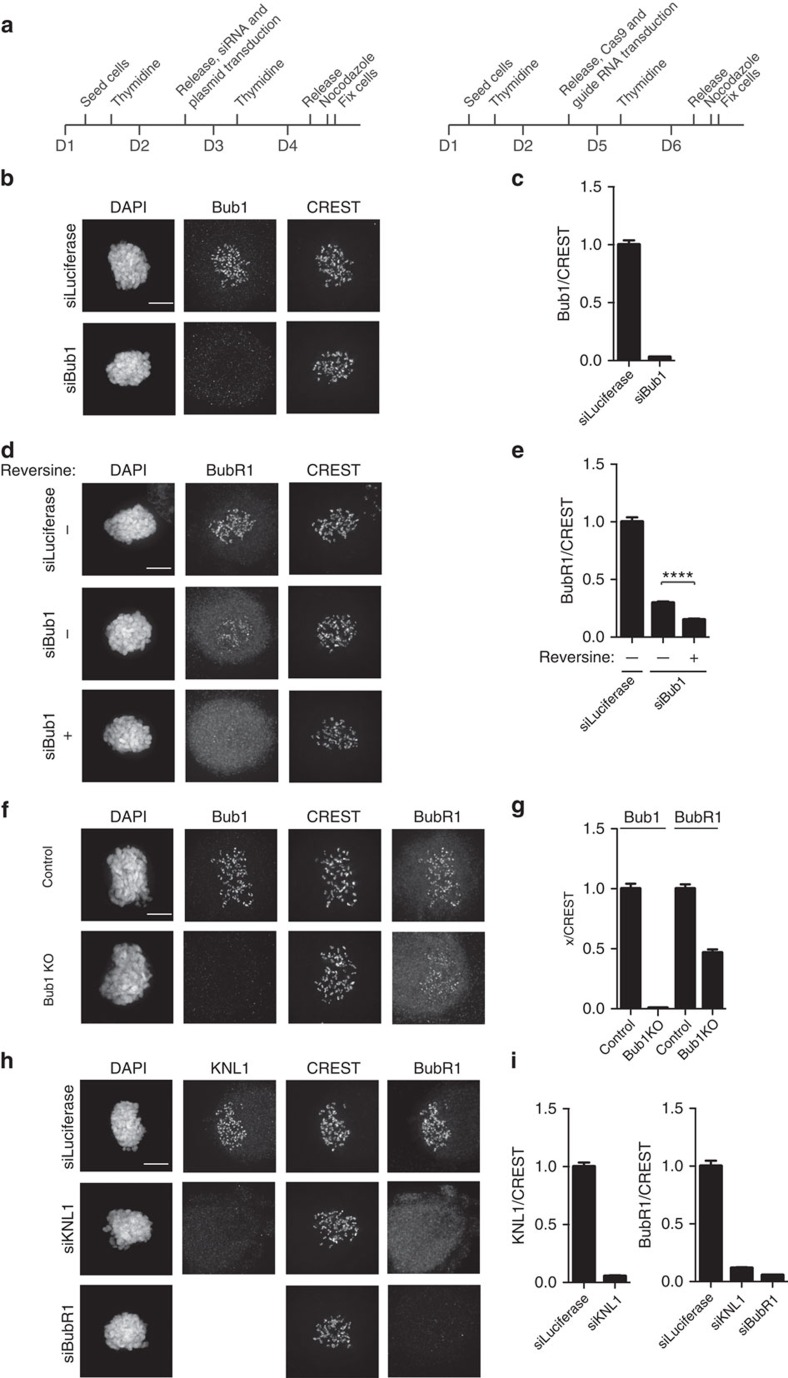
A Bub1-independent pool of BubR1 at kinetochores. (**a**) Outline of the synchronization protocol used in this study. Left, RNAi and rescue protocol; right, Bub1 CRISPR/Cas9 knockout protocol. (**b**) HeLa cells were treated with a control RNAi oligo (luciferase) or an RNAi oligo targeting Bub1 for 48 h and arrested in mitosis using nocodazole for 2 h. Cells were fixed and stained with 4,6-diamidino-2-phenylindole (DAPI), CREST (centromere marker) and Bub1 antibodies. (**c**,**e**,**g**,**i**) The kinetochore levels of Bub1, BubR1 and KNL1 were determined in the indicated conditions and normalized to CREST. An unpaired *t*-test was used for statistical comparison of the different samples in **e**. (****P≤0.0001). (**d**) HeLa cells were treated similarly as in **b** except reversine (0.5 μM) was added in one condition. (**f**) HeLa cells were transfected with Cas9 protein with guide RNA against Bub1 gene for 96 h and treated with nocodazole for 2 h. Cells were fixed and stained with DAPI, Bub1, CREST and BubR1 antibodies. (**h**) HeLa cells were treated with RNAi oligos against luciferase, KNL1 or BubR1 for 48 h and arrested by nocodazole for 2 h. Cells were fixed and stained with DAPI, KNL1, CREST and BubR1 antibodies. At least 160 individual kinetochores from eight cells were measured in each condition. The mean with s.e.m. is indicated. Scale bar, 5 μm.

We recently reported that a small region of Bub1 spanning residues 266–311 directly binds to BubR1 and facilitates the localization of BubR1/Bub3 to kinetochores^29^. Although RNA interference (RNAi) depletion of Bub1 by 95% strongly reduces BubR1 kinetochore levels, we always observe ∼25–35% of BubR1 remaining on kinetochores as also observed by others^28^,^30^ (Fig. 1a–e). Calibration of our BubR1 and Bub1 antibodies by immunofluorescence using RNAi-treated cells transfected with Venus–BubR1 or Bub1–Venus revealed that our antibodies were equally sensitive in detecting BubR1 and Bub1 at kinetochores (Supplementary Fig. 1A,B). We also measured the relative ratio of the two proteins in a mitotic cell extract using immunoprecipitated Venus–BubR1 and Bub1–Venus to normalize the respective antibodies to a green fluorescent protein (GFP) antibody by Li-cor quantitative western blotting. Using this approach, we detect very close to 1:1 protein levels of Bub1 and BubR1 (Supplementary Fig. 1C,D), which could be expected as the proteins arose by gene duplication^35^. Similar results were achieved when we knocked out the Bub1 gene in HeLa cells using a transient CRISPR/Cas9 knockout strategy. We observed a complete absence of Bub1 at kinetochores in 17% of the mitotic cells but these kinetochores still maintained ∼46% of BubR1 (Fig. 1a,f,g) (please note that the rabbit BubR1 antibody used for this gives a slightly higher level of BubR1 at kinetochores than the mouse monoclonal used throughout). These results argue that the BubR1 remaining at kinetochores after efficient Bub1 depletion is localizing independently of Bub1 potentially explaining the two kinetically different BubR1 pools observed by the Salmon lab^9^.

### Localization of BubR1/Bub3 by direct binding to MELTp

To determine how the Bub1-independent pool of BubR1/Bub3 is localizing, we focused on the outer kinetochore protein KNL1 that is known to play an important role in localizing checkpoint proteins. RNAi depletion of KNL1 by >90% reduced Bub1 kinetochore levels to the same extent as Bub1 RNAi^29^ but in contrast to Bub1 RNAi, KNL1 RNAi almost fully abolished BubR1 localization with only 5–10% remaining (Fig. 1h,i). Inhibiting the Mps1 checkpoint kinase with reversine^36^, in a Bub1 RNAi background, further reduced BubR1 kinetochore levels from 25–35% to ∼10% (Fig. 1d,e). Combined these experiments argue that Bub1-independent localization of BubR1/Bub3 is still dependent on KNL1 and Mps1. The most obvious explanation is that the BubR1/Bub3 complex binds directly to MELTp motifs in KNL1 and this would be in agreement with the previous observations that a KNL1 protein without active MELT repeats does not support BubR1/Bub3 kinetochore localization^20^,^21^.

**Figure 2:**
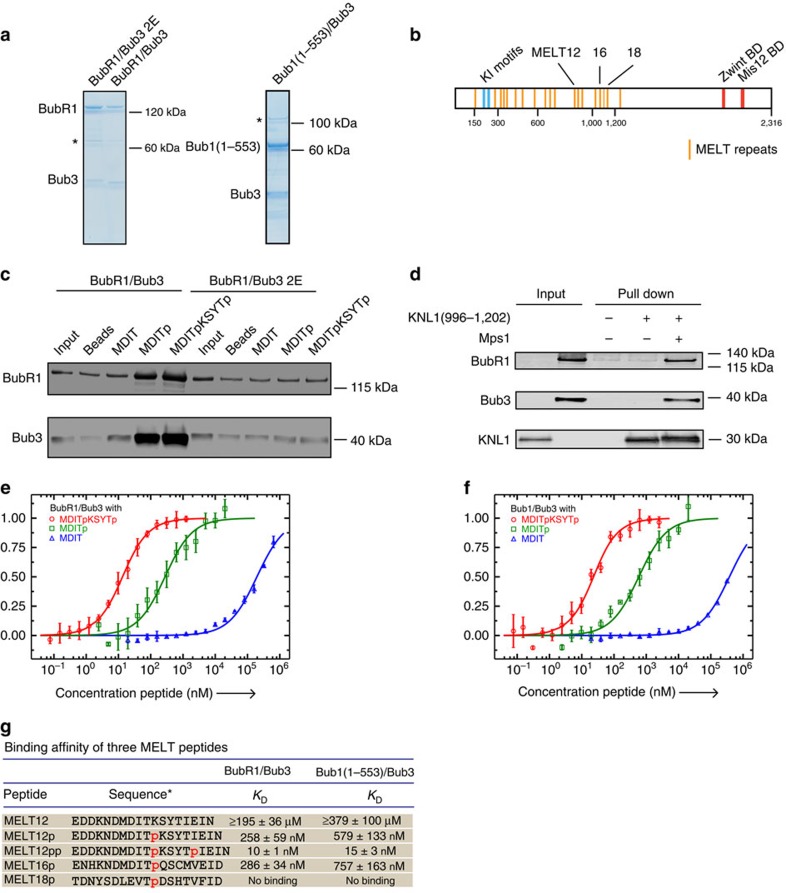
BubR1/Bub3 binds directly to phosphorylated MELT repeats. (**a**) Coomassie staining showing purified BubR1/Bub3 and BubR1/Bub3 2E (left), and purified Bub1(1–553)/Bub3 (right) from HEK293 cells. * indicates co-purifying contaminant. (**b**) Schematic of KNL1 and the position of MELT 12, 16 and 18. (**c**) Peptide-binding assay with purified BubR1/Bub3 and biotinylated MELT12 peptides was analysed by quantitative western blot with antibodies against BubR1 or Bub3. (**d**) KNL1 996–1,202 binding assay with purified BubR1/Bub3 analysed by quantitative western blot with antibodies against BubR1, Bub3 and KNL1. (**e**,**f**) Affinity measurement of BubR1/Bub3 (**e**) or Bub1(1–553)/Bub3 (**f**) to peptides (MELT12) with either unphosphorylated MDIT or single-phosphorylated MDITp or double-phosphorylated MDITpSYTp using MicroScale Thermophoresis (MST). Fraction bound versus the concentration of three MELT12 peptides are shown here. Data were derived from the ratio of the normalized time averaged (1 s) fluorescence intensities at defined time points of the MST traces (8 and −1 s, respectively). Lines represent fits of the data points using the law of mass action. The number of independent repeats was three for all measurements; error bars show the s.d. between these independent repeats. (**g**) Table showing *K*_D_ of BubR1/Bub3 and Bub1(1–553)/Bub3 for the different MELT12 peptides (data shown in **e**,**f**) and to single-phosphorylated MELT16 and 18. The *K*_D_ constants were obtained by fitting the fraction of bound protein to the quadratic solution of the binding reaction equilibrium derived from the law of mass action.

To confirm that the BubR1/Bub3 complex is indeed able to directly interact with MELTp, we reconstituted the complex by co-expressing FLAG–BubR1 and untagged Bub3 in HEK293 cells and following FLAG-affinity purification, the complex was further purified by size-exclusion chromatography (Fig. 2a). This purified complex contained <0.1% co-purified Bub1 as determined by quantitative western blot (Supplementary Fig. 1E). On streptavidin beads, we immobilized a biotinylated peptide encompassing MELT repeat 12 from KNL1 (Fig. 2b) in either its unphosphorylated form or a form where the MDIT was phosphorylated or one where both the MDIT and SYT were phosphorylated. The beads were then incubated with BubR1/Bub3 or with BubR1/Bub3 R202E/K222E (Bub3 R202E/K222E referred to as Bub3 2E from hereon), where key MELTp-interacting residues of Bub3 are mutated^26^. BubR1/Bub3 exclusively bound to the phosphorylated peptides, while BubR1/Bub3 2E did not bind (Fig. 2c; Supplementary Fig. 4). When both the MDIT and SYT were phosphorylated, we observed stronger binding. To test whether BubR1/Bub3 can bind phosphorylated KNL1 protein, we used a purified recombinant KNL1 fragment, which encompasses amino acids 996–1,202, that we have previously shown to be sufficient for KNL1 functionality^20^. This KNL1 fragment was first phosphorylated by Mps1 kinase and then immobilized on beads through a strep-tag. Purified BubR1/Bub3 bound to this KNL1 fragment only after treatment by Mps1 kinase (Fig. 2d).

We next wanted to quantitatively compare the ability of BubR1/Bub3 and Bub1/Bub3 to bind MELTp repeats. For this, we purified a FLAG–Bub1(1–553)/Bub3 complex in parallel with FLAG–BubR1/Bub3 (we could not obtain sufficient yields of the full-length Bub1/Bub3 complex, but have recently shown that Bub1 1–553 fully supports SAC signalling^29^). To determine the affinity of the complexes to MELTp repeats, we used MicroScale Thermophoresis (MST). Purified BubR1/Bub3 and Bub1(1–553)/Bub3 complexes were fluorescently labelled with maleimide chemistry for these measurements and tested for their ability to bind phosphorylated MELT12, MELT16 and MELT18 peptides (Fig. 2e–g; Supplementary Fig. 2). The binding of BubR1/Bub3 to MELT12 was strongly dependent on phosphorylation and the measured affinity was sensitive to the salt concentration in the buffer as expected for an interaction depending on electrostatic interactions with the phosphorylated threonine^26^ (Fig. 2e–g; Supplementary Fig. 2C). We conducted the affinity measurements at 50 mM NaCl as this produced the best quality data for the Bub1(1–553)/Bub3 complex. Under these conditions, both complexes bound to phosphorylated MELT12 and MELT16, but no binding was detected to phosphorylated MELT18. BubR1/Bub3 displayed slightly higher affinity for MELTp compared with Bub1(1–553)/Bub3 in these assays.

From this, we conclude that full-length BubR1/Bub3 can bind directly to MELTp repeats with an affinity that is sufficient to mediate physiological relevant interactions. The dynamics of the BubR1/Bub3 and Bub1/Bub3 interactions with MELTp might be different despite similar affinities and could explain the observed differences in KNL1 binding observed *in vivo*^28^. Thus, BubR1/Bub3 binds MELTp repeats in KNL1 independently of Bub1 and this facilitates kinetochore localization of 25–35% of BubR1 as a KNL1 protein without active MELT repeats recruits little BubR1/Bub3.

### Functional dissection of the two BubR1 kinetochore pools

Our data showed that two different pools of BubR1/Bub3 exist at kinetochores, but whether this reflected distinct functions was not clear. To determine this, we decided to separate the two pools using BubR1/Bub3 mutants and characterized their functions as described below.

**Figure 3:**
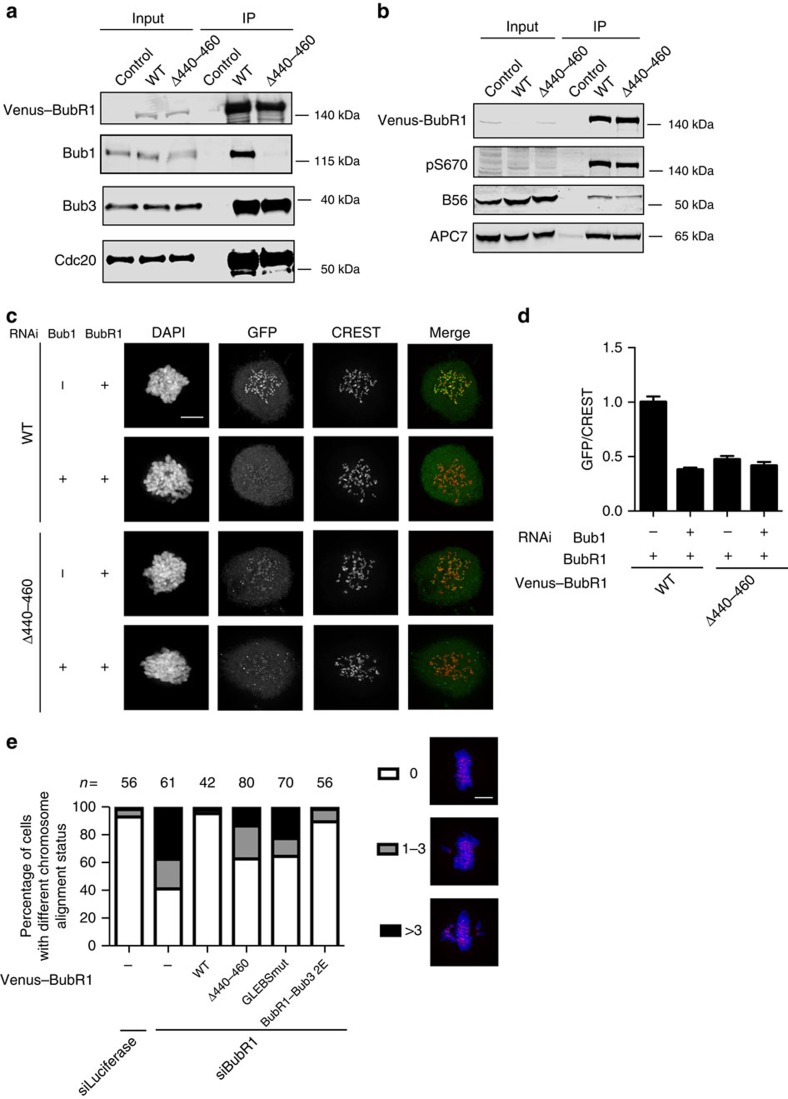
Bub1-dependent BubR1/Bub3 localization supports chromosome alignment. (**a**,**b**) Venus–BubR1 or Venus–BubR1Δ440–460 plasmid was transfected into HeLa cells for 48 h. After nocodazole treatment, mitotic cells were collected and lysed. Immunoprecipitation was performed against Venus tag by GFP-Trap beads. The presence of BubR1, Bub1, Bub3 and Cdc20 in **a**, and BubR1, BubR1 pS670, B56 and APC7 in **b** was analysed by Li-cor quantitative western blot. (**c**) RNAi-resistant Venus–BubR1 or Venus–BubR1Δ440–460 plasmid was co-transfected into HeLa cells with BubR1 RNAi oligo or both BubR1 and Bub1 RNAi oligos for 48 h. After nocodazole treatment, cells were fixed and stained with 4,6-diamidino-2-phenylindole (DAPI), GFP and CREST antibodies. Scale bar, 5 μm. (**d**) Kinetochore signals of BubR1 were determined and normalized to CREST. At least 160 individual kinetochores from eight cells were measured in each condition. The mean with s.e.m. is indicated. (**e**) HeLa cells were depleted of endogenous BubR1 and supplemented with RNAi-resistant Venus-tagged BubR1 WT, BubR1Δ440–460, BubR1 GLEBSmut or BubR1–Bub3 2E plasmid for 48 h. Cells were arrested in metaphase for 1 h by MG132 (10 nM) before fixation. Unaligned chromosomes outside the metaphase plate were counted in each condition and plotted. *n* indicates the number of cells counted in each condition. Images on the right are representatives of chromosome alignment defects observed.

Deletion analysis of a small fragment of BubR1, BubR1 350–483, that efficiently localizes to kinetochores^37^ revealed that the region from 440 to 460 was critical for BubR1 kinetochore localization (Supplementary Fig. 3B). Deletion of residues 440–460 from full-length BubR1 reduced its interaction with Bub1 almost a 100-fold based on quantitative western blot of immunopurifications of BubR1 complexes from nocodazole-arrested cells. Bub3 binding was not affected showing that residues 440–460 are specifically involved in Bub1 binding (Fig. 3a; Supplementary Fig. 3A). BubR1Δ440–460 was phosphorylated on Ser670, a phosphorylation that stimulates B56 binding, and maintained its ability to bind PP2A–B56, although the interaction was 50% reduced compared with BubR1 wild type (WT; Fig. 3b)^12^. We reasoned that BubR1Δ440–460 would mimic the Bub1-independent pool of BubR1. In immunofluorescence analysis of cells depleted of endogenous BubR1 but supplemented with short interfering RNA (siRNA)-resistant Venus-tagged BubR1Δ440–460, BubR1Δ440–460 still localized to kinetochores but this was insensitive to Bub1 RNAi. This further confirms a Bub1-independent pool of BubR1 (Fig. 3c,d). To analyse chromosome alignment, we depleted endogenous BubR1 and complemented with different RNAi-resistant BubR1 constructs. Following release from a thymidine block, the cells were treated with a proteasome inhibitor MG132 for 1 h and chromosome alignment was subsequently analysed by immunofluorescence. While BubR1 WT supported alignment, BubR1Δ440–460-complemented cells had severe chromosome alignment defects likely due to the reduced kinetochore levels of PP2A–B56 (Fig. 3e). In parallel, we generated another mutant BubR1–Bub3 2E (described in the next section), which mimics the Bub1-dependent pool of BubR1. In contrast to BubR1Δ440–460, this mutant almost fully supported chromosome alignment.

**Figure 4:**
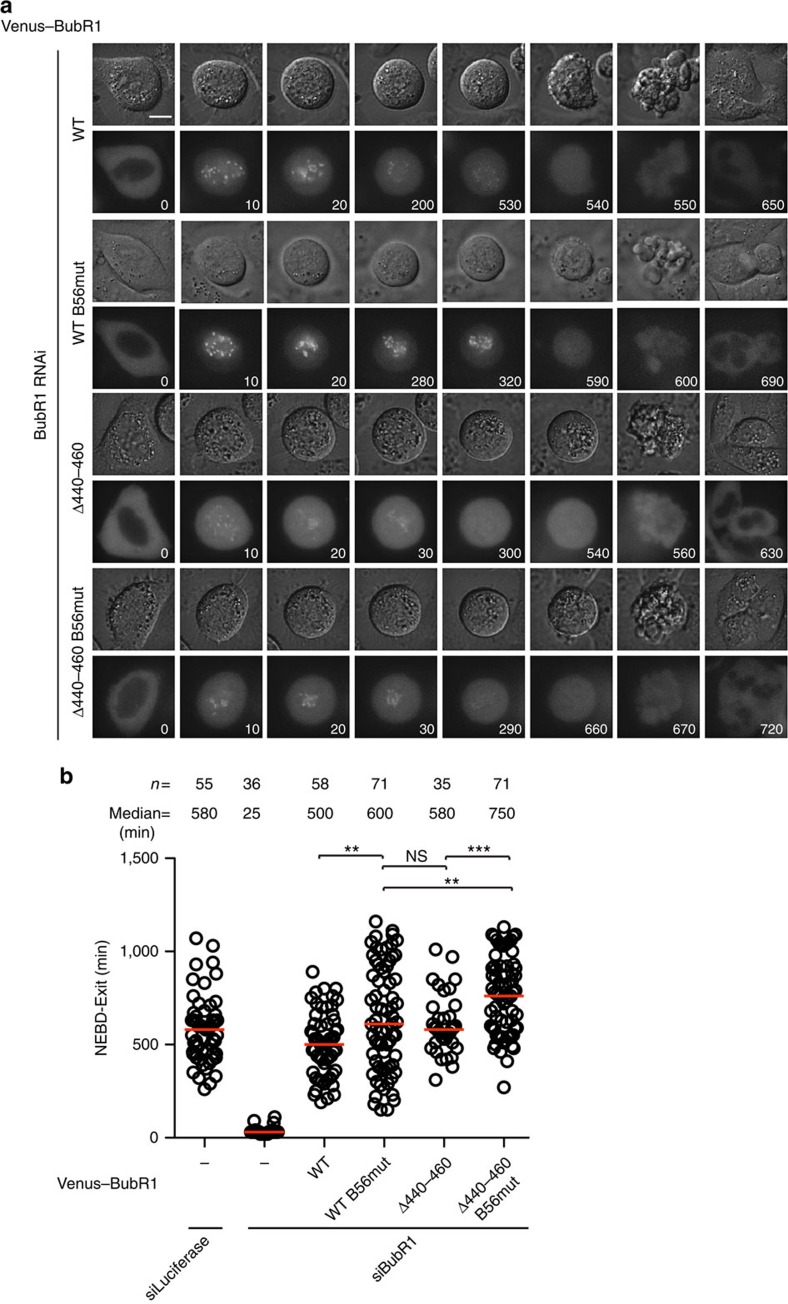
Time-lapse analysis of SAC proficiency of BubR1 mutants. (**a**) HeLa cells were depleted of endogenous BubR1 by RNAi and supplemented with RNAi-resistant Venus-tagged BubR1, BubR1 B56mut, BubR1Δ440–460 or BubR1Δ440–460 B56mut plasmid. Live-cell imaging was performed to record the time cells spent in mitosis after a treatment with low dose of nocodazole. Representative still images are shown. Scale bar, 5 μm. (**b**) Quantification of the live-cell imaging results of **a**. *n* indicates the total number of cells counted. Median is the median time cells spent in mitosis which is indicated by the red line in the plot. Each dot represents a single cell. A Mann–Whitney test was used for statistical comparison of the different samples. (***P≤0.001; **P≤0.01; NS, not significant).

To analyse the ability of BubR1 mutants to support checkpoint signalling, we depleted endogenous BubR1 and complemented with Venus-tagged siRNA-resistant BubR1 WT and mutants thereof. Using time-lapse microscopy, we measured the time from mitotic entry to exit in the presence of a low dose of nocodazole, a microtubule poison that strongly activates the SAC. In this assay, BubR1Δ440–460 fully supported checkpoint signalling, revealing that binding to Bub1 was not required for a functional checkpoint similar to a recent report from the Musacchio lab^28^ (Fig. 4a,b). Instead, preventing the interaction with Bub1 increased mitotic duration by 80 min compared with WT BubR1. To avoid any indirect effects from PP2A–B56 bound to BubR1, we mutated Leu669 and Ile672 to Ala in BubR1 (BubR1 B56mut) and BubR1Δ440–460, which abolishes B56 binding^12^. Mutation of the B56-binding site in BubR1 increased the time spent in mitosis by 100 min compared with BubR1 WT in this assay. Interestingly, mutation of the B56-binding site in BubR1Δ440–460 also increased mitotic duration showing that the Bub1-independent pool of BubR1 was sensitive to the SAC-silencing activity of BubR1 bound PP2A–B56 (refs 32, 33). Importantly, BubR1Δ440–460 B56mut still arrested 150 min longer than BubR1 B56mut, revealing a negative effect of the BubR1–Bub1 interaction on the SAC independently of PP2A–B56 (Fig. 4a,b).

Our results show that BubR1/Bub3 binding to Bub1 is required for chromosome alignment, but has a negative effect on the SAC.

### Kinetochore-localized BubR1/Bub3 stimulates SAC signalling

**Figure 5:**
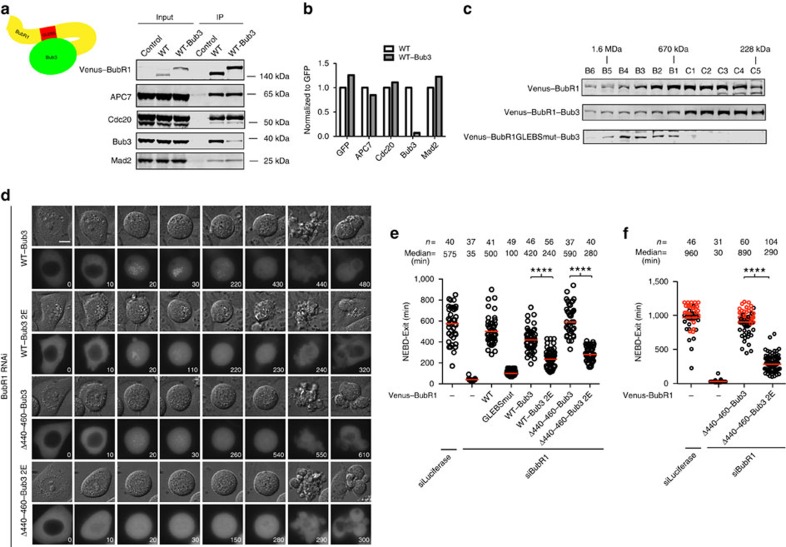
Kinetochore localization of the Bub1-independent pool of BubR1/Bub3 stimulates checkpoint signalling. (**a**) Cartoon of the internal binding between Bub3 and GLEBS domain on BubR1 in the fusion protein BubR1–Bub3. Venus-tagged RNAi-resistant wild-type (WT) BubR1 or BubR1–Bub3 plasmid was transfected into HeLa cells together with RNAi oligos against BubR1 for 48 h. After nocodazole treatment, mitotic cells were collected and lysed. Immunoprecipitation was performed against Venus tag by GFP-Trap beads. The presence of APC7, Bub3, Cdc20 and Mad2 was analysed by Li-cor quantitative western blot. (**b**) Western blot results in **a** were quantified and plotted. The values from BubR1 WT were set to 1. (**c**) Venus-tagged BubR1 WT or BubR1–Bub3 or BubR1 GLEBSmut–Bub3 plasmid was transfected into HeLa cells for 48 h. After nocodazole treatment, mitotic cells were collected and lysed and extract separated on a Superose-6 column and the relevant fractions analysed by western blot. Migration of molecular weight standards (670 kDa) or calculated molecular weights based on a calibration curve is indicated on top. (**d**) HeLa cells were co-transfected with BubR1 RNAi oligos and Venus-tagged BubR1–Bub3, BubR1–Bub3 2E, BubR1Δ440–460–Bub3 or BubR1Δ440–460–Bub3 2E plasmid. Live-cell imaging was performed to record the time cells spent in mitosis in the presence of low-dose nocodazole. Representative still images are shown. Scale bar, 5 μm. (**e**) Quantification of the live-cell imaging results of **d**. (**f**) HeLa cells were co-transfected with BubR1 RNAi oligos and Venus-tagged BubR1Δ440–460–Bub3 or BubR1Δ440–460–Bub3 2E plasmid. Live-cell imaging was performed to record the time cells spent in mitosis in the presence of taxol. Quantification of the live-cell imaging results is shown. *n* indicates the total number of cells counted. The red line in the plot indicates median time cells spent in mitosis. A Mann–Whitney test was used for statistical comparison of the different samples. (****P≤0.0001). Note: the red dots in **f** represent the cells still arrested in mitosis when filming stopped.

**Figure 6:**
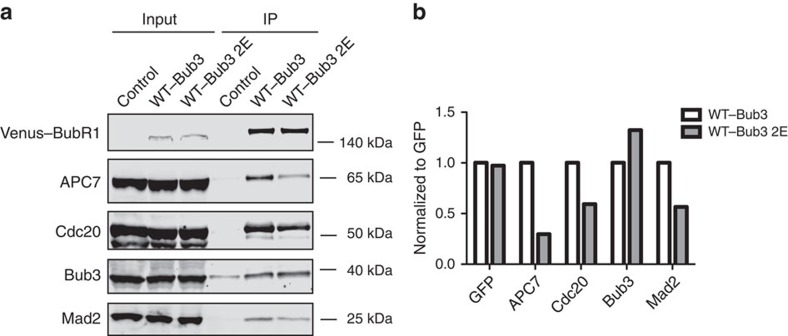
Preventing direct BubR1–MELTp binding reduces MCC formation. (**a**) HeLa cells were transfected with BubR1 RNAi oligos together with RNAi-resistant Venus-tagged BubR1–Bub3 or BubR1–Bub3 2E constructs for 48 h. After nocodazole treatment, mitotic cells were collected and lysed. Immunoprecipitation was performed against the Venus tag by GFP-Trap beads. The presence of APC7, Bub3 and Cdc20 and Mad2 was analysed by Li-cor quantitative western blot. (**b**) Western blot results were quantified and plotted. The signal from BubR1–Bub3 was set to 1.

From the above analysis, the Bub1-independent BubR1 mimicking mutant could still localize to kinetochores and fully support the SAC. To test whether the kinetochore localization of this mutant stimulated SAC signalling, we needed to specifically prevent BubR1/Bub3 interaction with MELTp. We observed that in-line fusion of Bub3 to the C terminus of BubR1 (referred to as BubR1–Bub3) strongly reduced binding of endogenous Bub3 by >90%, but not the kinetochore localization (Fig. 5a,b; Supplementary Fig. 3C,D). The most likely explanation is that the fused Bub3 efficiently binds to the GLEBS motif of BubR1 *in cis* hereby displacing endogenous Bub3. Indeed, size-exclusion chromatography revealed that a BubR1–Bub3 fusion protein eluted at a molecular weight of ∼400 kDa, while a similar fusion protein with a mutated GLEBS (E412K/E413K referred to as GLEBSmut) eluted at ∼1,000 kDa (Fig. 5c) supporting this idea. Using such a fusion protein would allow us to specifically interfere with MELTp interactions of Bub3 bound to BubR1 by introducing the Bub3 R202E/K222E. We therefore generated fusion protein BubR1–Bub3 and BubR1–Bub3 2E as well as BubR1Δ440–460–Bub3 and BubR1Δ440–460–Bub3 2E, and analysed their ability to support checkpoint signalling. We depleted endogenous BubR1 and then complemented cells with the different RNAi-resistant constructs and monitored mitotic duration in taxol or nocodazole using time-lapse microscopy. In taxol, the effect was marked in that BubR1Δ440–460–Bub3-expressing cells arrested for 890 min, while BubR1Δ440–460–Bub3 2E-expressing cells arrested for 290 min (Fig. 5f). In nocodazole, BubR1Δ440–460–Bub3 arrested for 590 min, while BubR1Δ440–460–Bub3 2E arrested for 280 min, arguing for a contribution of BubR1/Bub3 kinetochore localization under this condition (Fig. 5d,e). BubR1–Bub3 2E and BubR1Δ440–460–Bub3 2E supported checkpoint signalling better than BubR1GLEBSmut (median=100 min), but this is likely because some endogenous Bub3 still binds the fusion protein (Fig. 5a,b). Importantly, while BubR1Δ440–460–Bub3 still showed some kinetochore localization, although less than BubR1–Bub3, this was further reduced in BubR1Δ440–460–Bub3 2E (Fig. 5d; Supplementary Fig. 3C,D). These experiments therefore show that BubR1/Bub3 needs to bind directly to MELTp of KNL1 for efficient SAC signalling particular when few kinetochores are unattached. We analysed the ability of BubR1–Bub3 and BubR1–Bub3 2E to form MCC complexes by immunopurifying the fusion proteins from nocodazole-arrested cells. Indeed, mutating the MELTp-binding pocket of Bub3 in BubR1–Bub3 impaired the interaction with Mad2, Cdc20 and the APC/C, providing evidence that it is both the formation of the MCC and its interaction with the APC/C that are impaired (Fig. 6).

Importantly, BubR1–Bub3 2E is still able to localize to kinetochores through the interaction with Bub1 and this mutant supported chromosome alignment as mentioned above, showing that the two pools of BubR1–Bub3 combined ensures accurate chromosome segregation.

## Discussion

**Figure 7:**
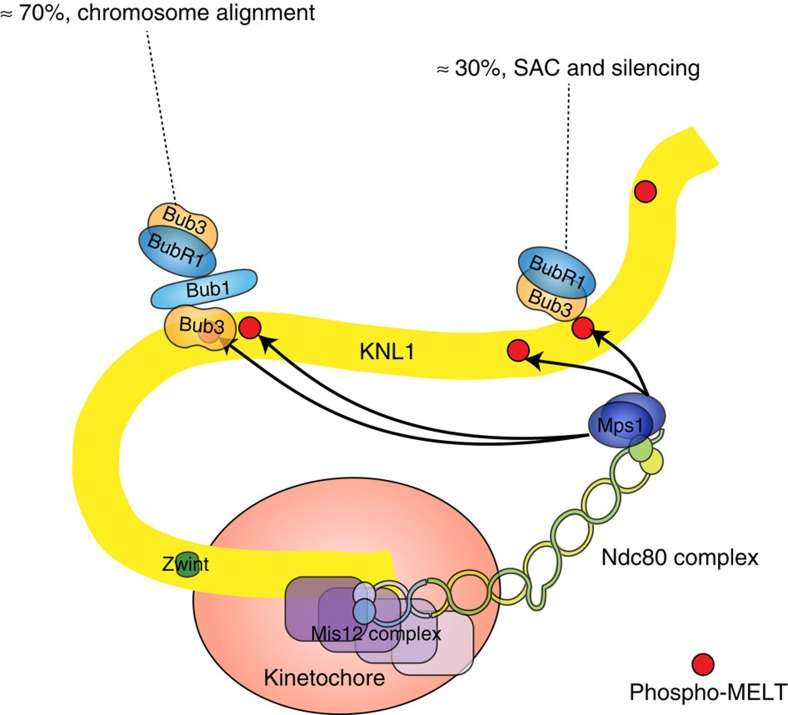
Model for the regulation of chromosome segregation by BubR1. The major population of BubR1/Bub3 depends on Bub1/Bub3 for localization and is required for alignment of chromosomes. The minor pool of BubR1/Bub3 interacts directly with phosphorylated MELT motifs on KNL1 and is required for SAC signalling and silencing. Bub3 bound to BubR1 is required for the localization of both populations.

Here we show that BubR1/Bub3 can localize independently of Bub1 to kinetochores by binding directly to MELTp repeats on KNL1. This conclusion is based on (1) a pool of BubR1 remains on kinetochores after efficient Bub1 removal or deletion of the Bub1 interaction motif in BubR1, (2) reconstituted full-length BubR1/Bub3 can bind MELTp repeats *in vitro* and localization *in vivo* in the absence of Bub1 still depends on Mps1 and KNL1, (3) mutation of the MELT binding pocket of Bub3 in the BubR1Δ440–460–Bub3 fusion protein impairs kinetochore localization, (4) BubR1Δ440–460 B56mut further enhances SAC due to its inability to dephosphorylate MELTp and (5) a KNL1 protein without MELT repeats is unable to recruit BubR1 to kinetochores^20^,^21^. The pool of BubR1/Bub3 localizing by directly binding to MELTp is required for efficient SAC signalling, particularly when few kinetochores are signalling, while the Bub1-dependent pool of BubR1/Bub3 is required for chromosome alignment but this pool does not interact with MELTp directly (Fig. 7). Whether the defect in chromosome alignment upon preventing the Bub1–BubR1 interaction is simply due to reduced levels of BubR1/Bub3 at kinetochores or there is a specific function of the Bub1-dependent BubR1/Bub3 pool is presently unclear. Since both pools of BubR1 binds to PP2A–B56, it can be that chromosome alignment only requires a certain amount of this phosphatase at kinetochores, which in theory could be provided by both pools of BubR1/Bub3. Alternatively, chromosome alignment might require more stable association of PP2A–B56 with kinetochores mediated by the Bub1-dependent pool of BubR1/Bub3.

It is important to point out that Bub3 is required for the localization of both pools of BubR1/Bub3 because Bub3 is required for the interaction between BubR1 and Bub1 (ref. 28), explaining the checkpoint and chromosome alignment defects of BubR1 mutants unable to bind Bub3. Our work thus provides a clear explanation for previous conflicting results on the role of BubR1/Bub3 at kinetochores with respect to SAC signalling. We do not favour that Bub3 as part of the BubR1/Bub3 complex has a major kinetochore-independent function downstream of MCC formation and indeed *in vitro* experiments have shown that BubR1 without Bub3 is a potent APC/C–Cdc20 inhibitor^7^,^34^,^38^.

How does direct BubR1/Bub3 binding to MELTp on KNL1 enhance checkpoint signalling? We favour that KNL1 acts to concentrate BubR1/Bub3 to bring it close to the site of Mad2–Cdc20 complex formation to facilitate efficient MCC formation at kinetochores. This could also explain the difference we observe between taxol and nocodazole. In taxol-arrested cells, few kinetochores are generating Mad2–Cdc20 complexes and therefore efficient SAC signalling depends more strongly on BubR1/Bub3 kinetochore localization. Because BubR1 mutants unable to bind Bub3 still have checkpoint activity, although strongly reduced, we do not envision that BubR1/Bub3 directly participates in the catalysis of Mad2–Cdc20 complexes. Indeed, Mad2 readily binds Cdc20 in the absence of BubR1 (refs 5, 37, 39). The stimulating effect of concentrating BubR1/Bub3 at kinetochores as part of the SAC might be bypassed by overexpressing BubR1 (ref. 31) and not needed in yeast due to its smaller size^40^,^41^.

Why does BubR1/Bub3 binding to Bub1 negatively influence the checkpoint? Although our affinity measurements with purified complexes and MELT peptides does not show major differences in affinity between BubR1/Bub3 and Bub1(1–553)/Bub3, we do not know the on and off rates of the complexes on MELTp and this is likely different based on the fluorescent recovery after photobleaching results from the Salmon lab^9^. It is possible that fast turnover of BubR1/Bub3 is needed for incorporation into the MCC or MCC diffusion. Second, BubR1 bound to Bub1 might not be able to incorporate into the MCC, potentially due to a steric clash between Bub1 and Cdc20 or Mad2. Indeed, Bub1 is absent from MCC complexes and mass spectrometry analysis of Bub1 complexes reveals the presence of BubR1, but no other MCC components^28^.

How is the distribution of BubR1 between the two kinetochore pools determined? At present, we do not know and several parameters need to be determined before we can address this. It is for instance unclear what the ratio of MELTp repeats to Bub1/Bub3 and BubR1/Bub3 is and whether there is any regulation of the Bub1–BubR1 interaction.

Our work underscores the complexity of the SAC and the multiple roles of BubR1 in ensuring proper chromosome alignment. It will be important in the future to understand if functionally distinct kinetochore pools of other checkpoint proteins exist and the role of these in generating the checkpoint signal.

## Methods

### Cell culture

HeLa cells (American Type Culture Collection) were cultivated in DMEM medium (Invitrogen) supplemented with 10% fetal bovine serum and antibiotics. Cells were synchronized with 2 mM thymidine for 24 h before co-transfection with siRNA oligos (100 nM as final concentration) and rescue constructs by Lipofectamine 2000 (Life Technologies). RNAi oligos targeting Bub1 (5′-GAGUGAUCACGAUUUCUAA-3′), KNL1 (5′-UUUCGUGGAUCCUUAAUCAGAUCUU-3′), BubR1 (5′-GAUGGUGAAUUGUGGAAUA-3′) and Luciferase (5′-CGUACGCGGAAUACUUCGA-3′) were used for RNAi depletions. Twelve hours after the transfection, the cells were arrested again by thymidine for another 24 h. The cells were released from thymidine and treated with nocodazole (200 ng ml^−1^) for 2 h when the majority of cells entered mitosis and fixed for immunofluorescence or challenged with low dose of nocodazole (30 ng ml^−1^) or taxol (200 nM) for live-cell imaging.

### Cloning

BubR1 complemetary DNA (cDNA) was cloned into pcDNA5/FRT/TO N-Venus vector by EcoRV and NotI sites with following PCR primers. Forward: 5′-TCGAGATATCGCGGCGGTGAAGAAG-3′ and reverse: 5′-GACTGCGGCCGCTCACTGAAAGAGCAAAGC-3′. Bub3 cDNA was cloned into pcDNA5/FRT/TO N-Venus using EcoRV and NotI sites with following PCR primers. Forward: 5′-CGATGATATCACCATGACCGGTTCTAACGAGTTC-3′ and reverse: 5′-CATAGCGGCCGCGCTCAAGTACATGGTGACTTGGG-3′. For making BubR1–Bub3 fusion constructs, BubR1 cDNA was inserted into Venus–Bub3 construct using EcoRV with following PCR primers. Forward: 5′-TCGAGATATCGCGGCGGTGAAGAAG-3′ and reverse: 5′-TCGAGATATCCTGAAAGAGCAAAGC-3′. For generating BubR1Δ440-460 construct, the following mutation primer was used. Forward: 5′-GTGCAGAGAAGAGAGCACAAGAAAGAACAGG-3′. For making Bub3 R202E, the following mutation primer was used. Forward: 5′-CTCTATTGAAGGCGAAGTGGCAGTTGAG-3′. For Bub3 K222E, the following mutation primer was used. Forward: 5′-GAAGTATGCCTTCGAATGTCACAGACTA-3′.

### Immunofluorescence and quantification

Cells growing on coverslips were washed once with PBS and fixed with 4% paraformaldehyde in PHEM buffer (60 mM PIPES, 25 mM HEPES, pH 6.9, 10 mM EGTA and 4 mM MgSO_4_) for 20 min at room temperature. Fixed cells were extracted with 0.5% Triton X-100 in PHEM buffer for 10 min. The antibodies used for cell staining include Bub1 (Abcam, ab54893, 1:400), BubR1 (made in house, 1:400), CREST (Antibodies Incorporated, 15-234, 1:400), GFP (Abcam, ab290, 1:400) and KNL1 (made in house, 1:200). All the fluorescent secondary antibodies are Alexa Fluor Dyes (Invitrogen, 1:1000). *Z*-stacks 200 nm apart was recorded on a Deltavision Elite microscope (GE Healthcare), using a × 100 oil objective followed by deconvolution using Softworx before quantification. Protein intensity on kinetochores was quantified by drawing a circle closely along the rod-like CREST staining covering the interested outer kinetochore protein staining on both ends. The intensity values from the peak three continuous stacks were subtracted of the background from neighbouring areas and averaged. The combined intensity was normalized against the combined CREST fluorescent intensity.

### Bub1 knockout by CRISPR/Cas9

Pre-designed DNA primers for Bub1 guide RNA template assembly were purchased from GeneArt (Thermo Fisher) with forward primer IVT-TAATACGACTCACTATAGTACAAGGGCAATGACC-T1 and reverse primer IVT-TTCTAGCTCTAAAACAGAGGGTCATTGCCCTTGT-T1. Guide RNA was synthesized according to the instruction using the GeneArt Precision gRNA Synthesis kit (Thermo Fisher). An amount of 625 ng of guide RNA and 2,500 ng of Cas9 nuclease (GeneArt Platinum Cas9 Nuclease, Thermo Fisher) were transfected into 4.5 × 10^5^ HeLa cells by Lipofectamine CRISPRMAX transfection reagent. Twenty-four hours later, the cells were split into a six-well plate with coverslips inside. Ninety-six hours after transfection, nocodazole (200 ng ml^−1^)-treated cells were fixed and immunofluorescence was performed as described above.

### Expression and purification of proteins

Ten 15-cm dishes were each seeded with 6 × 10^6^ HEK293 cells and the next day each transfected with 15 μg 3 × FLAG–BubR1 or 15 μg 3 × FLAG–Bub1(1–553)-expressing plasmid and 15 μg untagged Bub3-expressing plasmid using lipofectamine 2000 (Invitrogen). Forty-eight hours after transfection, the cells were collected by trypsination and cells washed in cold PBS with 1 mM phenylmethyl sulphonyl fluoride. The cells were resuspended in 5 ml buffer A (350 mM NaCl, 50 mM Tris·HCl (pH 8.0), 0.05% NP-40 and protease inhibitor cocktail (Roche)) and lysed using a nitrogen cavitation bomb. Lysate was clarified by centrifugation and then incubated with 300 μl FLAG-affinity resin (Sigma-Aldrich) for 90 min at 4 °C. The FLAG resin was washed with 20 ml buffer A and proteins eluted with three times 300 μl buffer A containing 500 ng ml^−1^ 3 × FLAG peptide (Sigma). The eluates were pooled and concentrated to 500 μl and loaded on a Superdex 200 10/300 GL column equilibrated with buffer B (150 mM NaCl, 25 mM HEPES·KOH (pH 7.8), 5% glycerol and 0.5 mM TCEP) and peak fractions eluting at ∼500 kDa pooled and concentrated using a vivaspin-4 concentration device. Proteins were stored at −80 °C.

His-Strep-KNL1 (996–1,202) was expressed in the *Escherichia coli* strain BL21(DE3) at 18 °C overnight and purified on a HisTrap column and subsequently on Strep-Tactin column. Finally, the protein was run on a Superdex 75 column in 150 mM NaCl, 50 mM NaP (pH 7.5), 10% glycerol and 0.5 mM TCEP, which resulted in a pure preparation eluting at the expected position.

### MELT peptides

The following peptides were purchased from biosyntan and checked by mass spectrometry to ensure they had the correct mass and were pure:

MELT12: Btn-PEG2-C-PEG2-EDDKND-Nle-DI-T-KSYTIEIN-amide

MELT12p: Btn-PEG2-C-PEG2-EDDKND-Nle-DI-Tp-KSYTIEIN-amide

MELT12pp: Btn-PEG2-C-PEG2-EDDKND-Nle-DI-Tp-KSYTpIEIN-amide

MELT16p: ENHKND-NIe-DI-Tp-QSC-NIe-VEIDY-amide

MELT18p: TDNYSDLEV-Tp-DSHTVFID-amide

Note: in MELT16, an extra Y was added to the c-terminal end for the quantification.

### MST measurements

MST experiments were performed on a NanoTemper Monolith NT.115 instrument with blue/red channels. Both complexes, BubR1/Bub3 and Bub1(1–553)/Bub3, were labelled using the Monolith NT Protein Labeling kit RED-MALEIMIDE (NanoTemper Technologies GmbH) according to the supplied protocol. The labelling efficiency was ∼90% and 75%, respectively, and it was estimated by independent measurements of the protein concentration combining spectroscopic measurements and a NT-647 red dye calibration curve in the corresponding MST interaction buffer. Samples were prepared in MST buffer (50 mM HEPES (pH 7.8), 200 mM NaCl, 0.5 mM TCEP, 0.05% Tween 20 or 50 mM Tris (pH 7.8), 50 mM NaCl, 0.5 mM TCEP and 0.05% Tween 20), loaded into premium coated capillaries and measurements were performed at 40% MST power. Laser off/on times were 5 and 30 s, respectively. The fluorescently labelled BubR1/Bub3 and Bub1(1–553)/Bub3 were used at concentrations of 25 and 12.5 nM, respectively, at 40% light-emitting diode power for all peptides except for the double-phosphorylated MELT12 peptide in MST buffer 50 mM Tris (pH 7.8), 50 mM NaCl, 0.5 mM TCEP and 0.05% Tween 20 that were performed at 4 nM concentration of the labelled proteins and 80% light-emitting diode power. The fluorescently labelled protein complexes were mixed with equivalent volumes (10 μl) of a twofold serial dilution of the different peptides starting at concentrations between 10 and 20 times the calculated *K*_D_. The fraction of bound protein was derived from the averaged (1 s averaged) normalized fluorescence signals at *t*=8 s of the labelled complexes at different ligand concentrations. The *K*_D_ constants were obtained by fitting the fraction of bound protein to the quadratic solution of the binding reaction equilibrium derived from the law of mass action (Equations 1 and 2), with the *K*_D_ being the single free parameter. The number of independent repeats was three for all measurements and error bars show the s.d. between these independent repeats.

For the binding event of L to P, the mass action law states:


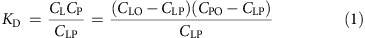


with

*K*_D_: dissociation constant

*C*_L_: free ligand concentration

*C*_P_: free protein concentration

*C*_LP_: bound complex concentration

*C*_LO_: total concentration of the ligand and

*C*_PO_: total concentration of the protein.

The fraction of bound protein yields:


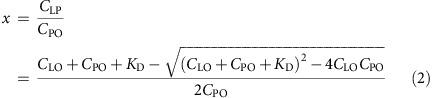


### Immunoprecipitation and protein binding assay

An amount of 2.5–7 μg of plasmid was transfected into HeLa cells in a 15-cm dish 48 h before collection. Nocodazole (200 ng ml^−1^)-arrested mitotic cells were shaken off the plates and lysed in lysis buffer containing 10 mM Tris·HCl, pH 7.4, 150 mM NaCl, 0.5 mM EDTA and 0.5% NP-40 with both protease and phosphatase inhibitors (Roche). After centrifugation at 17,000*g* for 10 min, the supernatant was applied to 20 μl of GFP-Trap beads (Chromotek) and shaken at 4 °C for 2 h. After three times of washing with 0.5 ml lysis buffer, the bound protein was eluted by boiling in 50 μl 2 × LDS loading buffer. Western blot was performed afterwards. The antibodies used for western blot include BubR1 (made in house, 1:1,000), Bub1 (Abcam, ab54893, 1:1,000), Bub3 (BD Transduction Lab, 611730, 1:500), GFP (Abcam, ab290, 1:2,000), APC7 (Bethyl, A302-551A, 1:2,000), Cdc20 (Santa Cruz, sc-13162, 1:1,000), Mad2 (Bethyl, A300-301A, 1:1,000) and KNL1 (made in house, 1:400).

For peptide-binding assays, 10 μg of peptide was diluted in 200 μl of PBS and applied to 30 μl of streptavidin beads (Thermo Scientific), which have been pre-washed with PBST (PBS with 0.1% Triton X-100). The mixture was shaken at room temperature for 1 h and the peptide–beads complex was resuspended in 0.5 ml binding buffer (50 mM Tris·HCl, pH 7.4, 150 mM NaCl and 0.3% Triton X-100 supplemented with both protease and phosphatase inhibitors). An amount of 2 μg of recombinant protein was applied to the peptide–beads complex and shaken at room temperature for 1 h. After four times of washing with binding buffer, bound protein was eluted by 50 μl of 2 × LDS buffer. Western blot was performed afterwards.

For BubR1/Bub3 and KNL1 recombinant protein binding assay, 0.4 μg of Mps1 protein (Invitrogen) was mixed with 8 μg of strep-tagged KNL1 (996–1,202) recombinant protein in 100 μl of reaction buffer containing 50 mM Tris·HCl, pH 7.4, 10 mM MgCl_2_, 0.5 mM dithiothreitol and 0.1 mM ATP. The mixture was incubated at 30 °C for 30 min for the phosphorylation reaction. Afterwards, the mixture was applied to 25 μl of Strep-Tactin beads (Qiagen), which was washed and resuspended in the binding buffer as used in peptide-binding assay. The whole mixture was shaken at room temperature for 1 h. After three times of washing, the beads were resuspended in 500 μl of binding buffer with 2 μg of the recombinant BubR1/Bub3 complex and shaken at room temperature for 1 h, followed by four times of washing. A volume of 25 μl of elution buffer containing 100 mM Tris·HCl, pH 8.0, 150 mM NaCl and 2.5 mM desthiobiotin was applied to the beads and the mixture was shaken at room temperature for 15 min. The eluted material was used for western blot analysis. Full scans of western blots are shown in Supplementary Fig. 3.

### Live-cell imaging

Live-cell imaging was performed on a Deltavision Elite system using a × 40 oil objective (GE Healthcare). Cells were transfected in a six-well plate and re-seeded in six-well Ibidi dishes (Ibidi) 1 day before the filming. Growth media was changed to Leibovitz's L-15 (Life technologies) before filming. Low dose of nocodazole (30 ng ml^−1^) or taxol (200 nM) was added into L-15 medium before filming started. Appropriate channels were recorded for 18–22 h and data were analysed using Softworx (GE Healthcare). Statistical analysis was carried out using Prism software.

### Data availability

The authors declare that all data supporting the findings of this study are available within the article and its Supplementary Information files.

## Additional information

**How to cite this article:** Zhang, G. *et al*. Two functionally distinct kinetochore pools of BubR1 ensure accurate chromosome segregation. *Nat. Commun.* 7:12256 doi: 10.1038/ncomms12256 (2016).

## Supplementary Material

Supplementary Information Supplementary Figures 1-4

## References

[b1] Lara-Gonzalez P., Westhorpe F. G. & Taylor S. S. The spindle assembly checkpoint. Curr. Biol. 22, R966–R980 (2012). 23174302 10.1016/j.cub.2012.10.006

[b2] Sacristan C. & Kops G. J. P. L. Joined at the hip: kinetochores, microtubules, and spindle assembly checkpoint signaling. Trends Cell Biol. 25, 21–28 (2015). 25220181 10.1016/j.tcb.2014.08.006

[b3] Pines J. Cubism and the cell cycle: the many faces of the APC/C. Nat. Rev. Mol. Cell Biol. 12, 427–438 (2011). 21633387 10.1038/nrm3132

[b4] Sudakin V., Chan G. K. & Yen T. J. Checkpoint inhibition of the APC/C in HeLa cells is mediated by a complex of BUBR1, BUB3, CDC20, and MAD2. J. Cell Biol. 154, 925–936 (2001). 11535616 10.1083/jcb.200102093 PMC2196190

[b5] Nilsson J., Yekezare M., Minshull J. & Pines J. The APC/C maintains the spindle assembly checkpoint by targeting Cdc20 for destruction. Nat. Cell Biol. 10, 1411–1420 (2008). 18997788 10.1038/ncb1799 PMC2635557

[b6] Han J. S. . Catalytic assembly of the mitotic checkpoint inhibitor BubR1-Cdc20 by a Mad2-induced functional switch in Cdc20. Mol. Cell 51, 92–104 (2013). 23791783 10.1016/j.molcel.2013.05.019 PMC3713096

[b7] Izawa D. & Pines J. The mitotic checkpoint complex binds a second CDC20 to inhibit active APC/C. Nature 517, 631–634 (2014). 25383541 10.1038/nature13911 PMC4312099

[b8] Howell B. J., Hoffman D. B., Fang G., Murray A. W. & Salmon E. D. Visualization of Mad2 dynamics at kinetochores, along spindle fibers, and at spindle poles in living cells. J. Cell Biol. 150, 1233–1250 (2000). 10995431 10.1083/jcb.150.6.1233 PMC2150717

[b9] Howell B. J. . Spindle checkpoint protein dynamics at kinetochores in living cells. Curr. Biol. 14, 953–964 (2004). 15182668 10.1016/j.cub.2004.05.053

[b10] Shah J. V. . Dynamics of centromere and kinetochore proteins; implications for checkpoint signaling and silencing. Curr. Biol. 14, 942–952 (2004). 15182667 10.1016/j.cub.2004.05.046

[b11] Lampson M. A. & Kapoor T. M. The human mitotic checkpoint protein BubR1 regulates chromosome-spindle attachments. Nat. Cell Biol. 7, 93–98 (2005). 15592459 10.1038/ncb1208

[b12] Kruse T. . Direct binding between BubR1 and B56-PP2A phosphatase complexes regulate mitotic progression. J. Cell. Sci. 126, 1086–1092 (2013). 23345399 10.1242/jcs.122481

[b13] Suijkerbuijk S. J. E., Vleugel M., Teixeira A. & Kops G. J. P. L. Integration of kinase and phosphatase activities by BUBR1 ensures formation of stable kinetochore-microtubule attachments. Dev. Cell 23, 745–755 (2012). 23079597 10.1016/j.devcel.2012.09.005

[b14] Foley E. A., Maldonado M. & Kapoor T. M. Formation of stable attachments between kinetochores and microtubules depends on the B56-PP2A phosphatase. Nat. Cell Biol. 13, 1265–1271 (2011). 21874008 10.1038/ncb2327 PMC3186838

[b15] Xu P., Raetz E. A., Kitagawa M., Virshup D. M. & Lee S. H. BUBR1 recruits PP2A via the B56 family of targeting subunits to promote chromosome congression. Biol. Open 2, 479–486 (2013). 23789096 10.1242/bio.20134051 PMC3654266

[b16] Taylor S. S., Ha E. & McKeon F. The human homologue of Bub3 is required for kinetochore localization of Bub1 and a Mad3/Bub1-related protein kinase. J. Cell Biol. 142, 1–11 (1998). 9660858 10.1083/jcb.142.1.1 PMC2133037

[b17] Lara-Gonzalez P., Scott M. I. F., Diez M., Sen O. & Taylor S. S. BubR1 blocks substrate recruitment to the APC/C in a KEN-box-dependent manner. J. Cell. Sci. 124, 4332–4345 (2011). 22193957 10.1242/jcs.094763 PMC3258114

[b18] Krenn V., Wehenkel A., Li X., Santaguida S. & Musacchio A. Structural analysis reveals features of the spindle checkpoint kinase Bub1-kinetochore subunit Knl1 interaction. J. Cell Biol. 196, 451–467 (2012). 22331848 10.1083/jcb.201110013 PMC3283998

[b19] Elowe S. . Uncoupling of the spindle-checkpoint and chromosome-congression functions of BubR1. J. Cell Sci. 123, 84–94 (2010). 20016069 10.1242/jcs.056507

[b20] Zhang G., Lischetti T. & Nilsson J. A minimal number of MELT repeats supports all functions of KNL1 in chromosome segregation. J. Cell Sci. 127, 871–884 (2014). 24363448 10.1242/jcs.139725

[b21] Vleugel M. . Arrayed BUB recruitment modules in the kinetochore scaffold KNL1 promote accurate chromosome segregation. J. Cell Biol. 203, 943–955 (2013). 24344183 10.1083/jcb.201307016 PMC3871444

[b22] Krenn V., Overlack K., Primorac I., van Gerwen S. & Musacchio A. KI motifs of human Knl1 enhance assembly of comprehensive spindle checkpoint complexes around MELT repeats. Curr. Biol. 24, 29–39 (2014). 24361068 10.1016/j.cub.2013.11.046

[b23] Yamagishi Y., Yang C.-H., Tanno Y. & Watanabe Y. MPS1/Mph1 phosphorylates the kinetochore protein KNL1/Spc7 to recruit SAC components. Nat. Cell Biol. 14, 746–752 (2012). 22660415 10.1038/ncb2515

[b24] Shepperd L. A. . Phosphodependent recruitment of Bub1 and Bub3 to Spc7/KNL1 by Mph1 kinase maintains the spindle checkpoint. Curr. Biol. 22, 891–899 (2012). 22521786 10.1016/j.cub.2012.03.051 PMC3780767

[b25] London N., Ceto S., Ranish J. A. & Biggins S. Phosphoregulation of Spc105 by Mps1 and PP1 regulates Bub1 localization to kinetochores. Curr. Biol. 22, 900–906 (2012). 22521787 10.1016/j.cub.2012.03.052 PMC3723133

[b26] Primorac I. . Bub3 reads phosphorylated MELT repeats to promote spindle assembly checkpoint signaling. Elife 2, e01030 (2013). 24066227 10.7554/eLife.01030 PMC3779320

[b27] Vleugel M. . Sequential multisite phospho-regulation of KNL1-BUB3 interfaces at mitotic kinetochores. Mol. Cell 57, 824–835 (2015). 25661489 10.1016/j.molcel.2014.12.036

[b28] Overlack K. . A molecular basis for the differential roles of Bub1 and BubR1 in the spindle assembly checkpoint. Elife 4, e05269 (2015). 25611342 10.7554/eLife.05269 PMC4337726

[b29] Zhang G., Lischetti T., Hayward D. G. & Nilsson J. Distinct domains in Bub1 localize RZZ and BubR1 to kinetochores to regulate the checkpoint. Nat. Commun. 6, 7162 (2015). 26031201 10.1038/ncomms8162 PMC4458899

[b30] Vleugel M. . Dissecting the roles of human BUB1 in the spindle assembly checkpoint. J. Cell. Sci. 128, 2975–2982 (2015). 26148513 10.1242/jcs.169821

[b31] Malureanu L. A. . BubR1 N terminus acts as a soluble inhibitor of cyclin B degradation by APC/C(Cdc20) in interphase. Dev. Cell 16, 118–131 (2009). 19154723 10.1016/j.devcel.2008.11.004 PMC2659634

[b32] Espert A. . PP2A-B56 opposes Mps1 phosphorylation of Knl1 and thereby promotes spindle assembly checkpoint silencing. J. Cell Biol. 206, 833–842 (2014). 25246613 10.1083/jcb.201406109 PMC4178970

[b33] Nijenhuis W., Vallardi G., Teixeira A., Kops G. J. P. L. & Saurin A. T. Negative feedback at kinetochores underlies a responsive spindle checkpoint signal. Nat. Cell Biol. 16, 1257–1264 (2014). 25402682 10.1038/ncb3065 PMC6485516

[b34] Han J. S., Vitre B., Fachinetti D. & Cleveland D. W. Bimodal activation of BubR1 by Bub3 sustains mitotic checkpoint signaling. Proc. Natl Acad. Sci. USA 111, E4185–E4193 (2014). 25246557 10.1073/pnas.1416277111 PMC4210015

[b35] Vleugel M., Hoogendoorn E., Snel B. & Kops G. J. P. L. Evolution and function of the mitotic checkpoint. Dev. Cell 23, 239–250 (2012). 22898774 10.1016/j.devcel.2012.06.013

[b36] Santaguida S., Tighe A., D'Alise A. M., Taylor S. S. & Musacchio A. Dissecting the role of MPS1 in chromosome biorientation and the spindle checkpoint through the small molecule inhibitor reversine. J. Cell Biol. 190, 73–87 (2010). 20624901 10.1083/jcb.201001036 PMC2911657

[b37] Lischetti T., Zhang G., Sedgwick G. G., Bolanos-Garcia V. M. & Nilsson J. The internal Cdc20 binding site in BubR1 facilitates both spindle assembly checkpoint signalling and silencing. Nat. Commun. 5, 5563 (2014). 25482201 10.1038/ncomms6563

[b38] Tang Z., Bharadwaj R., Li B. & Yu H. Mad2-Independent inhibition of APCCdc20 by the mitotic checkpoint protein BubR1. Dev. Cell 1, 227–237 (2001). 11702782 10.1016/s1534-5807(01)00019-3

[b39] Hardwick K. G., Johnston R. C., Smith D. L. & Murray A. W. MAD3 encodes a novel component of the spindle checkpoint which interacts with Bub3p, Cdc20p, and Mad2p. J. Cell Biol. 148, 871–882 (2000). 10704439 10.1083/jcb.148.5.871 PMC2174553

[b40] Vanoosthuyse V., Meadows J. C., van der Sar S. J. A., Millar J. B. A. & Hardwick K. G. Bub3p facilitates spindle checkpoint silencing in fission yeast. Mol. Biol. Cell 20, 5096–5105 (2009). 19846658 10.1091/mbc.E09-09-0762 PMC2793287

[b41] Windecker H., Langegger M., Heinrich S. & Hauf S. Bub1 and Bub3 promote the conversion from monopolar to bipolar chromosome attachment independently of shugoshin. EMBO Rep. 10, 1022–1028 (2009). 19680287 10.1038/embor.2009.183 PMC2728212

