# Supplementary material for: Two functionally distinct kinetochore pools of BubR1 ensure accurate chromosome segregation
Source: Nat Commun. 2016 Jul 26;7:12256. doi: 10.1038/ncomms12256 (PMC4963475; doi:10.1038/ncomms12256)
Supplement: Supplementary Information — Supplementary Figures 1-4 [file ncomms12256-s1.pdf]

# Supplementary figures and figure legends

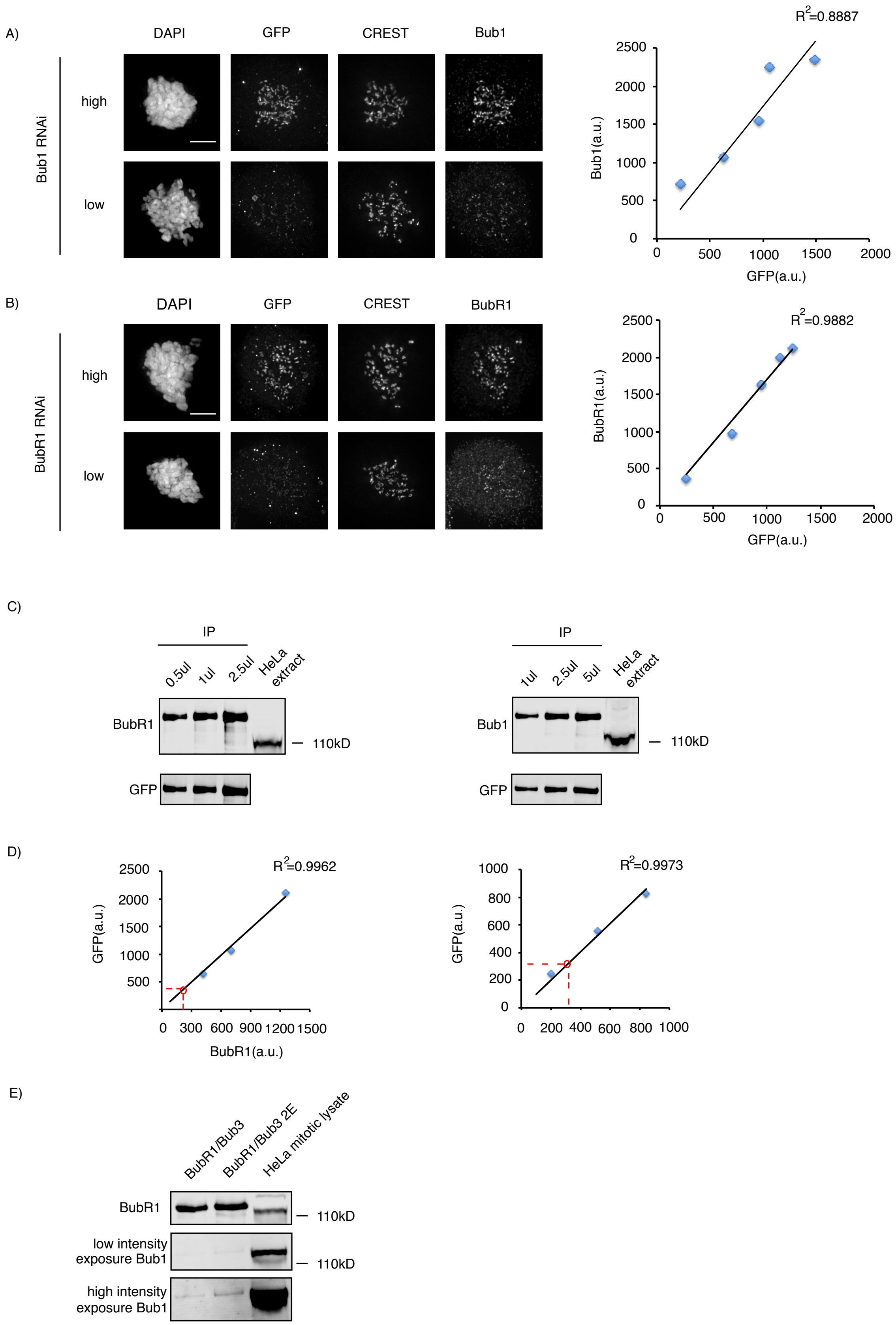


Zhang et al., Supplementary Figure 1

**Supplementary Figure 1. Equal amount of Bub1 and BubR1 in mitotic HeLa cells**

A-B) HeLa cells were co-transfected with Bub1 RNAi oligo and RNAi-resistant Bub1-Venus plasmid for 48 hours A) or with BubR1 RNAi oligo and RNAi-resistant Venus-BubR1 plasmid for 48 hours B). After 2 hours treatment of nocodazole, cells were fixed and stained with DAPI, CREST, GFP and Bub1 antibodies A) or DAPI, CREST, GFP and BubR1 antibodies B). Five cells expressing different levels of Bub1-Venus A) or Venus-BubR1 B) were recorded by immunofluorescence. Two cells expressing either relatively high level or low level of Bub1-Venus A) or Venus-BubR1 B) are shown here. Both GFP and Bub1 A) or BubR1 B) signals on kinetochores were measured and plotted. 100 individual kinetochores from five cells were measured in A,B). Scale bar, 5 µm. The mean with standard error of mean is indicated. C) HeLa cell lysate from nocodazole-arrested cells was analysed side by side with immunoprecipitated Venus-BubR1 or Bub1-Venus by BubR1, Bub1 antibodies and GFP antibody. The amount of Bub1 or BubR1 was measured by quantitative western blot and compared to GFP signal in each condition. D) Bub1 or BubR1 signal by Bub1 antibody or BubR1 antibody was plotted against the signal detected by GFP antibody. The amount of Bub1 or BubR1 in HeLa cell extract was measured and converted into GFP readings as shown by the red dotted line. Bub1 from HeLa lysate is equal to 317.99 units of GFP while BubR1 from same amount of lysate is equal to 330.38 units of GFP. So the ratio of Bub1/BubR1 is 0.962. E) The presence of Bub1 in the purified BubR1/Bub3 complex was analyzed side by side with cell lysate from mitotic HeLa cells by quantitative western blot against BubR1 or Bub1. The trace Bub1 could only be detected in the purified BubR1/Bub3 after high intensity exposure.

A)

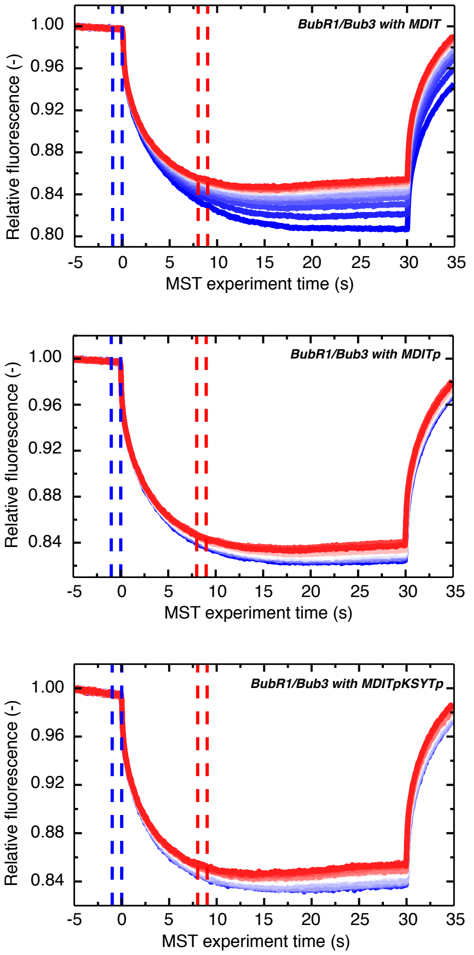


B)

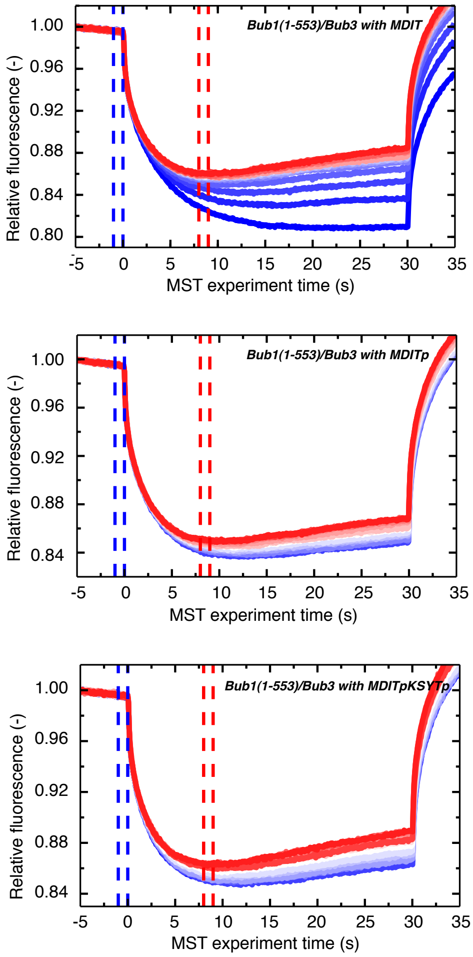


C)

Binding affinity of BubR1/Bub3 to MELT12 at different NaCl concentration

| Peptide | NaCl concentration | $K_D$ |
| --- | --- | --- |
| MELT12p | 0 mM | 82 ±14 nM |
| MELT12p | 50 mM | 259±59 nM |
| MELT12p | 150 mM | 379±56 nM |
| MELT12p | 200 mM | 936±117 nM |

D)

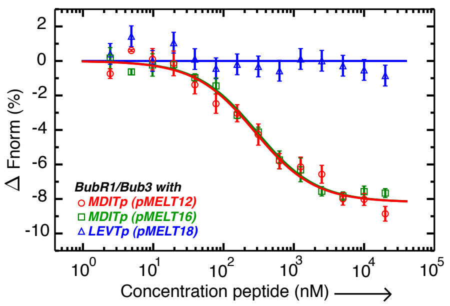


E)

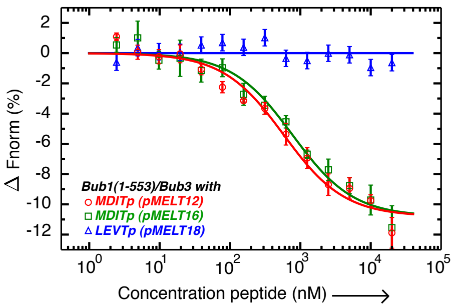

**Supplementary Figure 2. Affinity measurement of BubR1/Bub3 and Bub1(1-553)/Bub3 complexes to MELT peptides**

A-B) MST raw data of the affinity measurements of BubR1/Bub3 complex A) and Bub1(1-553)/Bub3 B) to unphosphorylated MDIT peptide, to single phosphorylated MDITp peptide and to double phosphorylated MDITpKSYTp. C) Binding affinity of BubR1/Bub3 complex to single phosphorylated MDITp at different salt concentrations. D-E) BubR1/Bub3 D) and Bub1(1-553)/Bub3 E) affinity measurement to single phosphorylated MELT12, 16 and 18 by MST. The difference in normalized time averaged fluorescence [‰] versus the concentration is shown here. Lines represent fits of the data points using the law of mass action (for MELT12 and 16) or the average of experimental data points (for MELT18). The error bars represent the standard deviation of each data point calculated from three independent thermophoresis experiments.

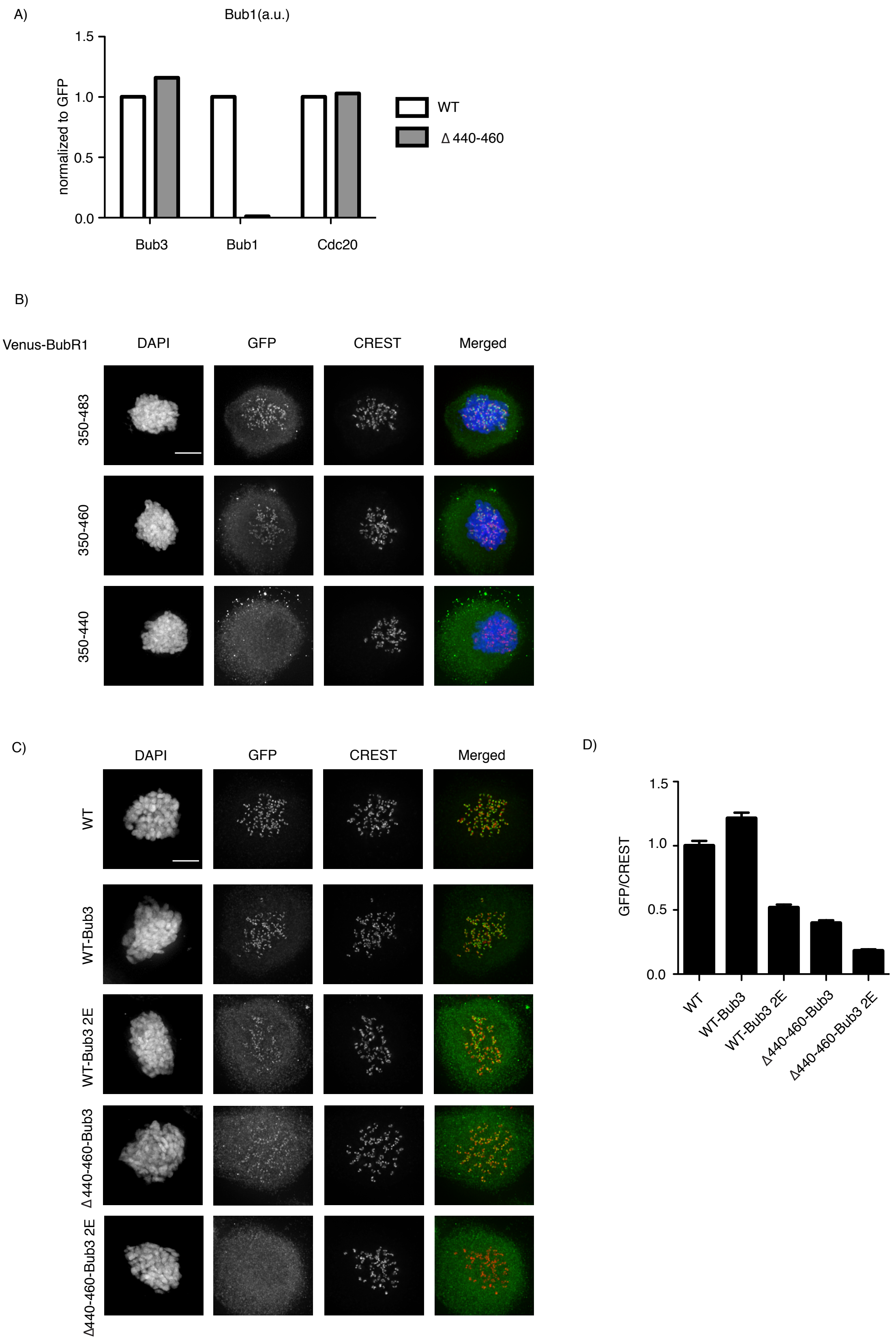


Zhang et al., Supplementary Figure 3

**Supplementary Figure 3. Localization of BubR1 proteins**

A) Western blot results in Fig 3A) were quantified and plotted. The values from BubR1 WT was set to 1 B) Venus-tagged BubR1 constructs encoding the region 350-483, 350-460 and 350-440 were transfected in HeLa cells for 48 hours. After two hours of nocodazole treatment, cells were fixed and stained by DAPI, GFP and CREST. C) HeLa cells were co-transfected with BubR1 RNAi oligos and with RNAi-resistant Venus-tagged BubR1, BubR1-Bub3, BubR1-Bub3 2E, BubR1Δ440-460-Bub3 or BubR1Δ440-460-Bub3 2E constructs for 48 hours. After two hours of nocodazole treatment, cells were fixed and stained for DAPI, GFP and CREST antibodies. Scale bar, 5 µm. D) Kinetochore signals of Venus were quantified from cells in C) and normalized to CREST. At least 160 individual kinetochores were measured from eight cells. Scale bar, 5 µm. The mean with standard error of mean is indicated.

Figure 2F)

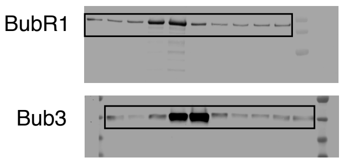


Figure 2G)

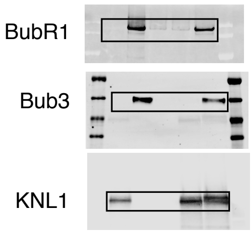


Figure 3A)

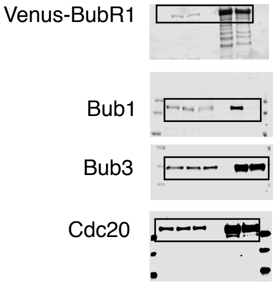


Figure 3F)

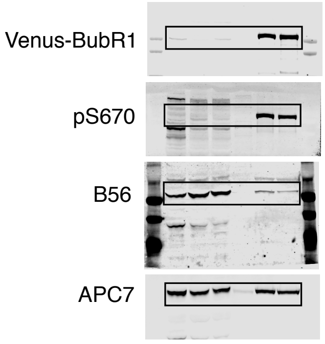


Figure 5A)

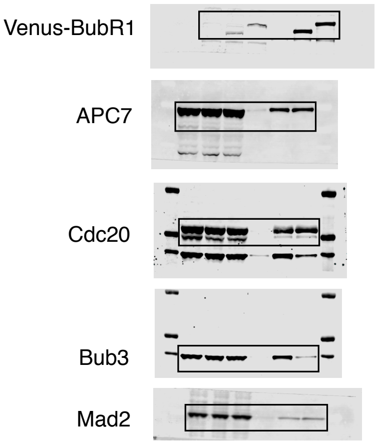


Figure 6A)

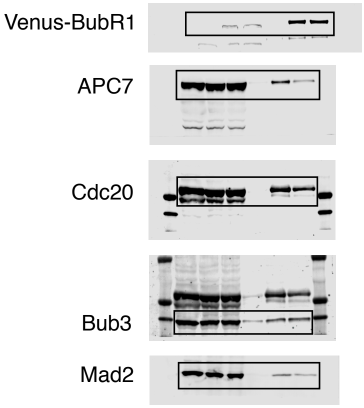

**Supplementary figure 4. Uncropped western blots**

Uncropped western blots and the cropped area indicated for main text figures.
